# Utilizing reactive oxygen species-scavenging nanoparticles for targeting oxidative stress in the treatment of ischemic stroke: A review

**DOI:** 10.1515/med-2024-1041

**Published:** 2024-11-19

**Authors:** Lingmin Shao, Can Wang, Gang Xu, Zewei Tu, Xinyuan Yu, Chao Weng, Jia Liu, Zhihong Jian

**Affiliations:** Department of Neurosurgery, Renmin Hospital of Wuhan University, Wuhan, 430060, Hubei, China; Department of Neurosurgery, Ezhou Central Hospital, Ezhou, 436000, Hubei, China; Department of Neurosurgery, Xiantao First People’s Hospital, Xiantao, 433000, Hubei, China; Department of Neurosurgery, Yale School of Medicine, New Haven, 06510, CT, United States of America; Department of Anesthesiology, Duke University Medical Center, Durham, 27710, NC, United States of America; Department of Neurology, Renmin Hospital of Wuhan University, Wuhan, 430060, Hubei, China

**Keywords:** oxidative stress, nanoparticles, ischemic stroke

## Abstract

Ischemic stroke, which accounts for the majority of stroke cases, triggers a complex series of pathophysiological events, prominently characterized by acute oxidative stress due to excessive production of reactive oxygen species (ROS). Oxidative stress plays a crucial role in driving cell death and inflammation in ischemic stroke, making it a significant target for therapeutic intervention. Nanomedicine presents an innovative approach to directly mitigate oxidative damage. This review consolidates existing knowledge on the role of oxidative stress in ischemic stroke and assesses the potential of various ROS-scavenging nanoparticles (NPs) as therapeutic agents. We explore the properties and mechanisms of metal, metal-oxide, and carbon-based NPs, emphasizing their catalytic activity and biocompatibility in scavenging free radicals and facilitating the delivery of therapeutic agents across the blood–brain barrier. Additionally, we address the challenges such as cytotoxicity, immunogenicity, and biodistribution that need to be overcome to translate these nanotechnologies from bench to bedside. The future of NP-based therapies for ischemic stroke holds promise, with the potential to enhance outcomes through targeted modulation of oxidative stress.

## 1 Introduction

Stroke is an acute and unexpected brain disease and remains one of the leading causes of death and disability worldwide. It is reported that the estimated global lifetime risk of stroke from the age of 25 years onward is 24.9% [1]. Ischemic stroke, which is triggered by a sudden decrease or loss of cerebral blood flow (CBF), accounts for 87% of all stroke cases, and poses a significant public health challenge [2].

Ischemic stroke is triggered by a sudden decrease or loss of CBF, leading to complex pathological and biochemical reactions. The reduction or cessation of CBF results in oxygen and glucose deprivation, a substantial decrease in adenosine triphosphate (ATP) levels, and a rapid influx of calcium, subsequently inducing the overproduction of reactive oxygen species (ROS), causing oxidative stress, and ultimately leading to irreversible cell damage or death [3]. Currently, the only United States Food and Drug Administration approved treatment for ischemic stroke involves clearing the thrombus to restore CBF, either through the administration of tissue plasminogen activator or mechanical thrombectomy. However, reperfusion, while restoring oxygen flow, also generates numerous ROS, which exacerbate brain injury [4,5]. Thus, oxidative stress is recognized as a major pathophysiological event in cerebral ischemia-reperfusion (IR) injury.

Despite significant progress in understanding the pathophysiology of ischemic stroke and advancements in comprehensive treatment strategies, antioxidant therapy in ischemic stroke has seen limited success. Although numerous neuro-antioxidative agents have shown effectiveness in *in vitro* models and preclinical studies, their translation into clinical practice has been unsuccessful due to low efficacy and/or deleterious side effects [6]. Therefore, there is a crucial need for developing novel brain drug delivery systems that specifically target oxidative stress in the treatment of ischemic stroke.

Nanomedicine, an emerging field that integrates chemistry, physics, biology, engineering, and medicine, offers great promise in delivering therapeutic agents to the brain. Utilizing materials at the atomic and molecular scale, nanotechnology provides innovative drug delivery systems capable of crossing the blood–brain barrier (BBB), thus offering new avenues for treating various brain diseases, including stroke. Recent studies suggest that antioxidant nanoparticle (NP) therapeutics could be a promising approach in the antioxidant therapy of ischemic stroke due to their unique features, such as small size, stability, and extended serum half-life [7]. This review briefly describes the role of oxidative stress in the pathophysiology of cerebral ischemic stroke, summarizes current literature on oxidative stress mechanisms, and discusses the application of antioxidant NPs in ischemic stroke treatment while outlining the challenges and prospects.

## 2 Overview of oxidative stress and ischemic stroke

### 2.1 Oxidative stress and its role in normal physiological and pathological conditions

Biological redox reaction is a fundamental biochemical reaction in the human body, which is also accompanied by the production of free radicals. The existence of free radicals in biological systems was first discovered in 1954 [8]. For several decades, free radicals were thought to bring exclusively damaging effects through oxidative modification, ultimately leading to irreversible dysfunction or even complete destruction. However, further studies show that they also have an essential role in physiological processes. For instance, nitric oxide (NO˙), a type of reactive nitrogen species (RNS), is crucial in vascular homeostasis. It acts as a signaling molecule that helps regulate blood pressure by promoting vasodilation, demonstrating how free radicals can have beneficial effects in the body [9].

Oxidative stress refers to the physiological and pathological reactions caused by the production of ROS and RNS in cells and tissues under harmful stimulation from the internal or external environment [10,11]. ROS are a series of natural byproducts generated in oxygen metabolism and comprised radicals like superoxide anion (O_2_˙^−^), hydroxyl radical (OH˙), and hydroperoxyl radical (HO_2_˙), together with non-radicals like hydrogen peroxide (H_2_O_2_) and hypochlorous acid. RNS is mainly composed of radicals like nitric oxide (NO˙), nitrogen dioxide radical (NOO˙), and non-radicals like peroxynitrite anion (ONOO‒). ROS and RNS are small molecules with unpaired electrons, making them highly reactive with biological micro-molecules.

Under normal physiological conditions, there is a balance in the oxidative–antioxidative system. Low or moderate oxidative stress activity serves as a basic protective mechanism essential for health. ROS and RNS are not always associated with deleterious effects. They also have critical functions in biological processes [12–14], such as cellular signaling, gene transcription regulation, and the control of cellular proliferation and differentiation, as well as in physiological processes [15–19], including cellular senescence, apoptosis, immune response, angiogenesis, vascular tone regulation, and the decomposition of toxic compounds. However, under pathological conditions, disturbances in the oxidative–antioxidative balance lead to a marked increase of ROS and RNS production, along with decreased oxidative defense. Oxidative stress is implicated in the pathogenesis of numerous acute and chronic diseases, such as acute myocardial infarction, stroke, cancer, hypertension, and neurodegenerative diseases [20–24]. Although the dual role of ROS and RNS in physiological and pathological conditions has been realized, some questions remain to be answered. The beneficial effects of ROS and RNS at low levels and their detrimental effects at high levels suggest that the concentrations of these reactive species may determine the shift. However, the exact concentrations triggering this shift are not generally known. Moreover, the possible contributing factors involved in the opposite actions are diverse, including cell types, duration of reactive species production, and the localization of their sources. The potential mechanisms for such phenomena are still unclear.

### 2.2 ROS/RNS generation in ischemic stroke

As a highly metabolically active organ, the brain relies on constant oxygen and glucose supply from the circulation. It has the highest rate of oxygen consumption in all organs, while the storage of energy within the brain is rather low. Although the brain accounts for only 2% of the body weight and never performs mechanical work, it requires about 20% of the body’s total oxygen supply. In addition, the brain has high concentrations of peroxidisable lipids and high levels of iron which act as pro-oxidants under oxidative stress but are accompanied by low levels of oxidative defense capacity. These characteristics make the brain more sensitive and vulnerable than other organs to oxidative stress. ROS and RNS are mainly produced by astrocytes and microglia in the brain. Cerebral IR initiates a cascade of molecular processes that are involved in the excess production of ROS and RNS via mitochondrial respiratory chain (MRC), nicotinamide adenine dinucleotide phosphate (NADPH)-oxidases (NADPH oxidases [NOXs]), xanthine oxidases (XOs), and nitric oxide synthases (NOS) [25–29].

#### 2.2.1 MRC

The mitochondrial electron transport chain is the primary source of ROS [30]. It has been found that at least seven sites in mitochondria partially contribute to the generation of ROS [31–33]. In normal physiological conditions, mitochondria reduces O_2_ to H_2_O by cytochrome c oxidase in Complex IV of the electron transport chain, and only 0.1–2% of O_2_ is reduced by the mitochondria to generate ROS [34].

In cellular respiration, a small amount of O_2_˙^−^ is produced as a byproduct of ATP generation by the oxidative phosphorylation process of MRC. Before leaving the mitochondria, it is converted into H_2_O_2_ by superoxide dismutase (SOD) and then acts as an intracellular messenger in the nervous system [35]. However, in ischemic conditions, hypoxia interrupts the oxidative phosphorylation process of MRC. Oxygen gets exhausted before the glucose and mitochondria make a switch to the anaerobic glycolytic pathway of ATP production. The anaerobic glycolysis leads to the accumulation of lactic acid and H^+^ which subsequently results in acidosis. The acidic environment further promotes the production of reactive species by providing H^+^ for the conversion of O_2_˙^−^ into other types of ROS, such as H_2_O_2_ or the more reactive OH˙ [36]. A further study showed that the accumulation of succinate during ischemia was found to be a potential mitochondrial metabolite that drives excessive ROS production [37].

During the reperfusion phase after the ischemic interruption, the recovery of MRC also leads to a large increase in the production of mitochondrial ROS. The reversal of complex I of the MRC appears to be a significant contributor, and as such MRC attenuation at complex I has been proposed as a potential strategy [38,39]. Mitochondrial complex I is a key enzyme in cellular energy metabolism and has been recognized as one of the main sources of ROS in neurons and astrocytes [40]. Complex I-related ROS has been linked to the oxidative damage occurring during ischemia/reperfusion [41]. Recent studies have further demonstrated the importance of complex I. It is found that the slow transition of complex I from the active (A) form to the deactivated dormant (D) form takes place during ischemia in the brain and complex I in the D-form serves as a protective mechanism preventing the oxidative burst upon reperfusion [42–45]. In a neonatal mice model subjected to cerebral hypoxia-ischemia (HI) and reperfusion, HI changed the conformation of complex I from A-form into the D-form and reperfusion rapidly converted the D-form into the A-form and increased ROS generation; however, administration of S-nitrosating agent decelerated the D to A transition, attenuated oxidative stress, and improved neurological recovery [39]. Understanding the A/D transition of mitochondrial complex I may contribute to the development of new therapeutic interventions for cerebral IR injury.

#### 2.2.2 NOXs

NOXs are another important source of ROS generation in cerebral ischemia especially in the following reperfusion injury. NOXs are multicomponent enzymes containing catalytic NOX subunits that generate superoxide by transporting electrons across the cell membrane from NADPH to oxygen molecules [46,47]. In physiological conditions, NOX enzymes work normally as membrane-bound enzymes that produce ROS for biological functions such as blood pressure control and microbial killing. However, in pathological situations, NOXs contribute significantly to oxidative stress injury from superoxide overproduction and ROS imbalance. NOX1 to NOX5, dual oxidase 1 and 2 are the seven NOX family members that have been identified. Of the NOX isoforms, NOX1, NOX2, and NOX4 have been detected in different regions of the brain, including intracranial vessels and neuronal tissues [48]. After ischemic stroke, the expression of NOX2 and NOX4 was shown to be increased in microglia, neurons, and endothelial cells [49–51]. NOX2 is the major contributor to N‐methyl‐d‐aspartate receptor-triggered superoxide generation during ischemic stroke [52]. In mice, both NOX1 and NOX2 knockout decreased the size of stroke lesions [53–55]. Additionally, NOX4 knockout protected the brain from oxidative damage after stroke [51]. Hence, these NOX isoforms present a potential target in stroke therapy.

#### 2.2.3 XO

XO is also considered to be a source of ROS generation during ischemic stroke. XO is a molybdo-flavin enzyme that catalyzes the oxidation of hypoxanthine to xanthine as well as the oxidation of xanthine to uric acid [56]. There are two interconvertible forms of this enzyme, xanthine dehydrogenase (XDH; NAD‐dependent dehydrogenase) and XO (oxygen‐dependent superoxide production oxidase) [57]. XDH is the predominant type under nonmonic conditions. Ischemia causes the catabolization of cellular ATP into hypoxanthine, which accumulates in the ischemic tissue, and XDH is simultaneously cleaved to the active form of XO. After that, during the reperfusion phase, the activity of XO increased. It oxidizes the reactions of hypoxanthine to xanthine and xanthine to uric acid, thus resulting in the production of O_2_˙^−^ and H_2_O_2_ [4,28].

#### 2.2.4 NOSs

NOS is involved in RNS generation in ischemic stroke. The common RNS in cerebral IR injury includes NO˙, NOO˙, and ONOO‒. NO˙ is generated as a byproduct of the amino acid l-arginine metabolism. l-arginine is converted into l-citrulline and NO˙ via a 5-electron oxidation of a guanidine nitrogen of l-arginine, which is carried out by the enzymes known as NOS [58]. The latter two are both produced by NO˙, which is released as a vasodilator by endothelial cells during reperfusion. Three isoforms of NOS have been identified in the neuronal system, including endothelial NOS (eNOS), neuronal NOS (nNOS), and inducible NOS (iNOS) [59]. eNOS is activated in the early stage of ischemia, producing a modest quantity of NO and therefore maintaining the CBF. However, the over-activation of nNOS and iNOS leads to a high amount of RNS generation in focal ischemia and the following reperfusion [60,61], which may reach toxic levels, inhibit the MRC, and participate in the inflammatory and cytotoxic actions that contribute to neuronal death [29,62].

### 2.3 Role of oxidative stress in the pathophysiology of cerebral ischemic stroke

**Figure 1:**
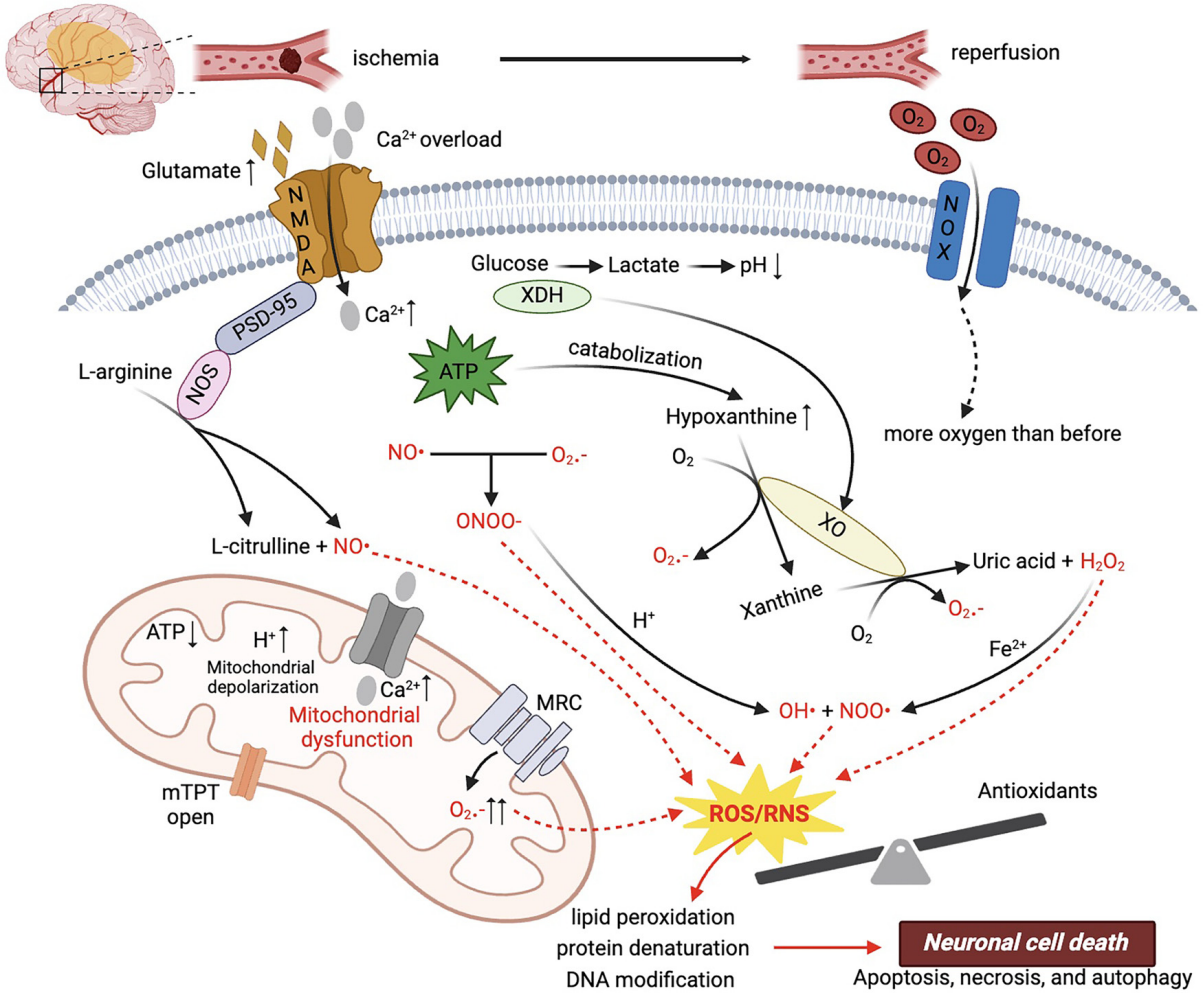
Mechanism of oxidative stress in IR injury.

As a consequence of the overproduction of ROS and RNS, oxidative stress is considered to be a major pathophysiological event in cerebral IR injury. Acute ischemic stroke attack and the following reperfusion injury cause a series of pathophysiological changes. After cerebral ischemia, the reduced blood supply causes energy failure and lactate acidosis. A shortage of oxygen and glucose delivery makes the energy metabolism of mitochondria convert to anaerobic metabolism, following a decrease in ATP level and loss of ionic homeostasis in neurons [63,64]. The failure to maintain ionic gradients leads to depolarization of the neuronal membrane and subsequent activation of a variety of ionic channels such as sodium and calcium channels, which results in the excessive release of glutamate [65]. Glutamate is a major neurotransmitter regulating a variety of excitatory synapses, but excessive glutamate causes excitotoxicity. Oxidative stress is a pathological phenomenon tightly linked to glutamate-mediated excitotoxicity. There are increasing evidence showing the essential role of oxidative stress in the pathophysiology of ischemic stroke. The excessive production of ROS and RNS has detrimental effects on neurons, glial cells, and vascular endothelial cells, including lipid peroxidation, protein denaturation, and DNA modification as well as fragmentation [10]. It also has a great responsibility in the progression of post-stroke reperfusion injury by activating inflammation, apoptosis, and autophagy pathways [66,67]. Inflammatory cascades accompany the oxidative stress attacks on neural tissues, leading to apoptosis via tanglesome pathways including p38 MAPK, p53, ERK1/2, and Keap1–Nrf2 pathway [68–72]. Additionally, autophagy-related signaling pathways have been shown to be significantly activated in neurons, glial cells, and brain microvascular cells during cerebral ischemia [73]. They are mediated by an enormous number of unfolded proteins produced by endoplasmic reticulum stress, excitotoxicity-induced NMDA receptor activation, intracellular calcium overload, and overproduction of ROS due to mitochondrial malfunction, as well as an excessive RNS level [74–77]. Furthermore, a link between autophagy and inflammation in ischemic stroke has been discovered as well, with evidence showing that inflammation directly triggers autophagy [78]. Therefore, as a part of the complex cascade reactions triggered by IR injury, oxidative stress plays a critical role in the pathophysiology of ischemic stroke and presents a potential target in stroke therapy (Figure 1).

## 3 Application of ROS-scavenging NPs in the treatment of ischemic stroke

**Figure 2:**
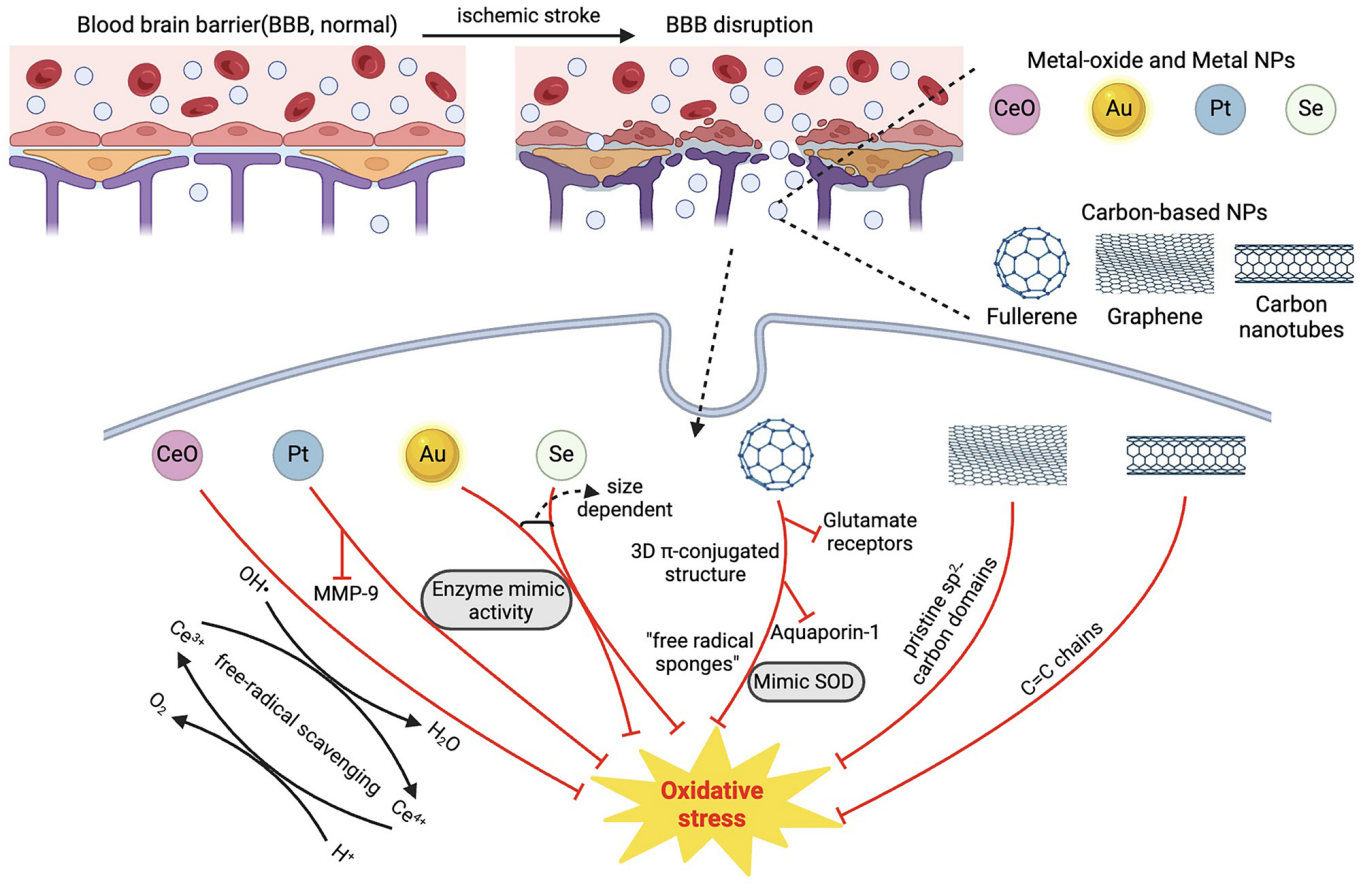
Utilizing ROSscavenging NPs for the treatment of ischemic stroke.

The endogenous enzymatic and non-enzymatic antioxidant defense system plays a vital role in maintaining the oxidative–antioxidative balance. Furthermore, the exogenous antioxidants also contribute significantly to antioxidative stress by targeting various cellular signaling pathways and subsequently increasing the level of endogenous antioxidant defenses. As promising antioxidants are being identified, the next challenge is how to deliver these therapeutic agents to target oxidative stress and reduce cerebral IR injury. The rapidly developing nanotechnology offers a bright future for overcoming the issues associated with pharmaceutical therapy for ischemic stroke. Nanoscale materials with unique physicochemical properties, such as small size, surface chemistry, high surface-to-volume ratio, and the potential for targeted delivery to brain tissues by passing through the BBB, make them ideal candidates for biomedical applications [79]. The NPs-based drug design has also been shown to improve drug pharmacokinetics, pharmacodynamics, and safety, and prevent off-target interactions [80]. Several varieties of ROS-scavenging NPs have been developed to validate the targeting of oxidative stress, and these NPs have made considerable improvements against cerebral IR injury. Increasing studies show that some NPs can imitate the capabilities of exogenous antioxidant enzymes to suppress cell apoptosis and improve cell survival following cerebral I/R injury [81]. The outer surface of NPs has higher ratios of active electrons due to their large surface-to-volume ratio, leading to an increase in their catalytic activity [82,83]. There are several varieties of NPs that have been exploited as potential biologically active antioxidants because of their redox property, including metal and metal-oxide NPs and carbon-based NPs (CbNPs) (Figure 2).

### 3.1 Metal and metal-oxide NPs

Metallic NPs are non-toxic and biocompatible, and the free electrons on the surface enable them to show strong ROS-scavenging activity, such as cerium oxide NPs (CeONPs), platinum NPs (PtNPs), gold NPs (AuNPs), and selenium NPs (SeNPs).

Cerium oxide is a metal oxide with potential redox activity, due to the rapid changes between Ce^4+^ and Ce^3+^ [84]. The reduction of Ce^4+^ to Ce^3+^ leaves oxygen vacancies in the lattice. Furthermore, Ce^3+^ interacts with ˙OH to produce Ce^4+^, which is then converted to Ce^3+^ and O_2_ by H^+^ [85]. Due to the oxygen vacancies on the surface, CeONPs can redox cycle between a Ce^4+^ and Ce^3+^ bulk state [86]. The oxygen buffering property allows CeONPs to exert their catalytic activities which imitate the free-radical scavenging properties of SOD and catalase in reducing the intracellular ROS and improving cells survival under oxidative stress [87]. Moreover, the synthesis of ultrasmall CeONPs (3 nm) increased the ratio of Ce^3+^ in NPs to approximately 57% and the higher ratio of Ce^3+^ improves the catalytic properties of CeONPs [88]. CeONPs provided a strong and stable protection for cardiac progenitor cells in the *in vitro* model of cardiac ischemia [89]. In addition, administration of europium-doped CeONPs limited ROS accumulation and ameliorated intestinal IR injury [90]. In a mild traumatic brain injury model, CeONPs improve neuronal survival and cognitive function by preserving endogenous antioxidant systems and decreasing macromolecular free radical damage [91]. CeONPs synthesized with aminocaproic acid also showed promising results against subarachnoid hemorrhage via potent antioxidative, neuroprotective, and anti-inflammatory activities [88]. Similarly, CeONPs showed great potential in ischemic stroke treatment [92,93]. CeONPs reduce approximately 50% of ischemic cell death in the mouse hippocampal slice model of cerebral ischemia and the neuroprotective effect was due to a modest reduction in ROS [94]. Other modifications of CeONPs, such as bioactive zeolitic imidazolate framework-8 and edaravone-loaded CeONPs also reduced the oxidative damage and apoptosis of neurons in ischemic stroke [95,96].

PtNPs are widely used in cosmetics due to their antioxidant properties. Researchers found that PtNPs can mimic the activity of antioxidant enzymes (peroxidase, SOD, and catalase), scavenge free radicals, and convert O_2_˙^−^ into H_2_O and O^2^ [97–99]. Due to the antioxidant capabilities of PtNPs *in vitro*, they have been used in research for the treatment of IR injury. For example, the administration of PtNPs before liver IR injury decreased the ROS levels and protected hepatic tissue against oxidative damage in a mouse model [100]. In a study conducted by Takamiya et al. [101], a 2–3 nm Pt nanoplatform showed satisfactory neuroprotective effects following transient middle cerebral artery occlusion treatment, as evidenced by reduced infarct volume and enhanced neurovascular unit production through deactivating matrix protease MMP-9.

AuNPs have been researched and exploited in a variety of biological applications due to their inertness and resistance to surface oxidation [102–104]. AuNPs can paradoxically exhibit either oxidative or antioxidant activity in biological systems, depending on their size. Small AuNPs (2 nm) significantly enhanced helium-based cold atmospheric plasma-induced apoptosis by decreasing the intracellular glutathione which led to the generation of intracellular ROS, while 40 and 100 nm AuNPs failed to enhance cell death [105]. Administration of 20 nm AuNPs showed protective effects in both oxygen–glucose deprivation/reperfusion and focal cerebral IR injury model of rats, while opposite effects were observed for 5 nm AuNPs [106,107]. In addition, AuNPs show enzyme-mimic activities, such as peroxidase, glucose oxidase, SOD, and catalase [108–111]. These enzyme-like activities allow them to react with superoxide and hydrogen peroxide to detoxify ROS. However, controlling the physicochemical properties of the AuNPs remains the first obstacle for endeavoring real-life applications [111].

SeNPs are highly bioavailable, low-dispersed bioactive compounds with strong antioxidant properties [112]. Because of their promising therapeutic effect, SeNPs are utilized in the research for the treatment of ischemic stroke. SeNPs improve the functional properties of neurons and astrocytes and contribute to their survival by regulating the antioxidant system, cellular metabolism, and inflammatory reactions accompanying ischemic damage. Investigations demonstrate that SeNPs can inhibit necrosis and greatly reduced apoptosis in the primary culture of mouse neurons and astrocytes during oxygen–glucose deprivation [113,114]. In a mouse model of ischemic stroke, it was found that SeNPs were transferred to the brain via transferrin receptor-mediated endocytosis, then decreased the neuroinflammation, and increased the survival of hippocampal neurons [115]. The cytoprotective effects of SeNPs are size-dependent, which can be arranged in descending order: 100 nm > 400 nm > 50 nm [116]. Furthermore, the protective effects of SeNPs in ischemic stroke are mediated by the activation of the Ca^2+^ signaling system of astrocytes and reactive astrogliosis [117].

### 3.2 CbNPsP

CbNPs are becoming attractive due to their unique properties related to the quantum confinement of the electron’s movement at discrete energy levels in the nanometric structure. The existence of heteroatoms in chemically modified nanocarbon can lead to the generation of ROS. However, CbNPs may also exhibit ROS scavenging effects. CbNPs include an extensive spectrum of structures from zero-dimensional structures (0D) to three-dimensional structures (3D), of which the most researched allotropes are fullerene (0D), carbon nanotube (CNT) (1D), graphene (2D), and graphite (3D) [118]. Each member of the carbon family exhibits unique properties and has been extensively utilized in a range of applications, from drug delivery to imaging, diagnosis, and disease therapy. CbNPs have received extensive attention for their potential in the study of ischemic stroke due to their antioxidative and redox regulation functions.

Fullerene is one of the allotropes of carbon which usually exists as C60 NPs. It has a distinctive spherical structure and an abundance of conjugated double bonds, offering the potential for simple and extensive surface decoration in biomedical applications [119,120]. The antioxidant capacity of fullerene relies on its proficiency in electron absorption and subsequent dispersion through its 3D π-conjugated structure, which is extensively distributed across its surface [121]. The unique surface chemistry makes it highly receptive to the radical species and capable of absorbing electrons, effectively acting as “free radical sponges” for these highly reactive species. Due to this catalytic property, fullerene can function as SOD and scavenge free radicals. Despite their potential to engage in SOD-like activity, which could theoretically result in elevated H_2_O_2_ levels, the coordinated action of fullerenes in scavenging superoxide anion and H_2_O_2_ does not trigger an augmentation in H_2_O_2_ production. In addition, modification of fullerene, such as carboxy fullerene and polyhydroxylated fullerene, can enhance the stability and facilitate their localization in mitochondria, leading to the reduction of free radical generation. Carboxy fullerene can protect against excitatory necrosis and neuronal apoptosis [122]. Vani et al. found that fullerene had a protective effect on cerebral infarction and inhibited nitrosative and oxidative stresses in a rat model of ischemic stroke [123]. Fullerenol, which is a polyhydroxylated derivative of C60 fullerene, is an effective scavenger of free radicals [124]. It can reduce ischemic brain injury and edema by alleviating oxidative damage. The neuroprotective effect of fullerenol is exerted by the blockade of glutamate receptors, reduction of intracellular calcium levels, and inhibition of aquaporin-1 expression [125]. Furthermore, Hsieh et al. found that different surface functional groups of fullerene had distinct effects on the regulation of oxygen metabolism in target cells, potentially inducing or reducing the generation of ROS [126]. The antioxidant activity of fullerene may be related to its size, structure, and surface chemical properties.

Graphene is a two-dimensional material composed of coplanar carbon atoms and arranged in a hexagonal lattice pattern with sp^2^ hybridization. Due to its unique electron mobility, thermal conductivity, and biocompatibility, there has been a growing interest in the use of graphene-based nanomaterials (GBNs) in nanomedicine over the past few decades [127,128]. GBNs show strong activity against hydroxyl radicals and modest activity against hydrogen peroxide, lipid peroxyl radicals, and stable radicals [129,130]. The principal members of the GBNs include single-layer graphene, bilayer graphene, multilayer graphene, graphene oxide (GO), reduced graphene oxide (rGO), and chemically modified graphene. Producing defect-free single-layer graphene is a challenging task due to its highly reactive surface and the difficulty of suspending it in water. As a result, for biological applications, GO and rGO are the preferred materials due to their ability to address this challenge [131]. Both GO and rGO have been investigated for their potential use in the therapy of stroke. Kim et al. investigated the antioxidant mechanism of GO flakes based on their protective effect against ROS-mediated mortality of implanted mesenchymal stem cells following myocardial infarction. The researchers discovered that GO flakes provided a platform for mesenchymal stem cell adhesion and inhibited a series of detrimental cell-signaling cascades, which led to the anoikis of MSCs in response to ROS [132]. Mendonça et al.’s study found that rGO could penetrate the thalamus and hippocampus of rats through systemic injection. This entry of rGO involved a transient decrease in the paracellular tightness of the BBB, as evidenced by the extravasation of Evan’s Blue stain into the brain [133]. Importantly, the rGO-induced temporary opening of the BBB did not seem to cause significant adverse effects. While a stroke can disrupt the BBB, the extent or duration of this disruption cannot be controlled. However, the temporary permeabilization of the BBB caused by rGO may be intentionally leveraged to improve the brain’s uptake of delivery systems for diagnostic or therapeutic purposes. Thus, rGO may be used to create a controlled therapeutic window for delivering drugs to the ischemic site. In a recent study, a facile CO-release platform was developed for the treatment of stroke, based on the size-dependent adsorption properties of ruthenium carbonyl clusters (Ru-carbon monoxide [CO]) onto GO. The release of CO was induced by photothermal therapy, which oxidized RuII(CO)_2_ to RuO_2_ on the GO surface. To demonstrate the vasodilation and stroke protective effect of the RuO_2_/RuII(CO)_2_/6Ru–CO–GO composite, a cortical photothrombotic ischemia rat model was employed. The results showed a decrease in infarct volume in the group treated with the RuO_2_/RuII(CO)_2_/6Ru–CO–GO composite, suggesting its potential as a stroke treatment. Although there have been promising developments, there are still some important issues to be solved before clinical application. First, the graphene product family such as GO and rGO have very different characteristics, and a standardization protocol needs to be developed to distinguish and characterize different molecules [134]. Moreover, the *in vivo* degradation of the graphene family needs to be improved. While some research suggests that a majority of graphene can be excreted from the body through urine, there is still a notable amount that remains in organs for over 270 days [135].

CNTs are cylindrical-shaped nanostructures composed of carbon atoms. CNTs can exist in single or multi-layered forms, referred to as single-walled and multi-walled CNTs (MWCNTs). These unique structures possess exceptional chemical, mechanical, and electrical properties, making them valuable tools in nanomedicine. Similar to carotenoids, studies found that the C═C chains on the structure of CNTs have a ROS-scavenging effect. MWCNTs have been shown to prevent the oxidation of materials such as polystyrene, polyethylene, and polypropylene, although their effect is not as strong as that of phenolic antioxidants [136]. Despite the acute oxidative effect of PEGylated single-wall CNTs on rat hippocampus, their long-term effect, 1 week after injection, was increased expression of antioxidant enzyme genes, enhanced antioxidant defense, and decreased ROS production [137]. Additionally, amine-modified single-walled CNTs have been shown to provide neuroprotection to rats after ischemic stroke and benefit behavioral functions. Despite their benefits, CNTs still have limitations that hinder their use, including poor solubility in water, low biodegradability and dispersivity, and the potential for deleterious drug-induced oxidative stress and lung disease [138–140].

## 4 Challenges and future directions

The application of antioxidant NPs has garnered significant interest in the treatment of ischemic stroke. However, there are some biocompatibility and safety concerns to consider. Potential toxicity, immunogenicity, and the persistent presence of NPs in the body are concerns that need rigorous investigation. NPs have the potential to interact with various cellular compounds, leading to cytotoxic effects that disrupt cell balance. These adverse effects are intricately linked to the NPs’ size, shape, and surface characteristics. The size of NPs plays a crucial role in determining their cytotoxicity. Smaller NPNPs have a higher surface area-to-volume ratio, enabling them to interact with numerous cellular chemicals, which amplifies their toxic effects. Both the core material and the surface coating need to be biocompatible and safe for interaction with biological tissues. For instance, 5 nm AuNPs exhibit a propensity to induce oxidative stress, particularly as AuNPs with smaller diameters tend to accumulate in the nucleus and organelles, ultimately causing DNA damage [106,141]. These cytotoxic effects present a significant obstacle to the widespread clinical utilization of NPs.

In addition, antioxidant NPs may trigger immune responses or inflammation when introduced into the body, potentially exacerbating the damage caused by ischemic stroke. Depending on their surface properties and composition, they can polarize immune cells toward an anti-inflammatory or pro-inflammatory phenotype [142–144]. Strategies to mitigate immunogenicity should be explored, such as surface modifications to minimize recognition by immune cells or incorporation of immunosuppressive agents within the NPs.

Moreover, another concern is the potential for NPs to accumulate in specific organs or tissues, leading to long-term toxicity. NPs are susceptible to clearance by the mononuclear phagocytic system, predominantly through the actions of phagocytic cells in the liver and spleen. This clearance process can potentially result in damage to the respective organs. They can trigger the adsorption of complement proteins and antibodies onto their surfaces in the bloodstream, forming a “corona.” This corona then serves as a signal for immune cell membrane receptors, leading to the initiation of phagocytosis [145]. This phenomenon ultimately reduces the exposure of drugs and their ability to penetrate the brain, consequently causing the accumulation of nanomaterials in organs other than the brain. Understanding the biodistribution of NPs in the body is essential to minimize the risk of the persistent presence of NPs in the body. The precise role and function of NPs in other organs necessitate thorough evaluation, particularly as strategies to mitigate off-target effects are crucial for the clinical application of nanomaterials. These matters remain largely unresolved and warrant further in-depth investigation.

In summary, while antioxidant NPs hold significant promise for treating ischemic stroke, addressing their biocompatibility, cytotoxicity, and biodistribution challenges is crucial. Future research must focus on optimizing NP design to enhance their therapeutic efficacy while minimizing potential risks, ultimately paving the way for safer and more effective clinical applications.

## 5 Conclusions

The exploration of ROSs-scavenging NPs has opened new avenues for addressing the intricate challenges of ischemic stroke treatment. Metal, metal-oxide, and CbNPs have demonstrated substantial potential in scavenging deleterious ROS and ameliorating the effects of cerebral IR injury. However, while these NPs offer innovative therapeutic strategies, their translation into clinical practice is encumbered by considerable challenges. The cytotoxicity and immunogenicity associated with NPs, along with concerns about their long-term presence and biodistribution in the body, require meticulous examination and resolution. Advances in nanotechnology must continue to refine the size, shape, and surface properties of these NPs to optimize their therapeutic effects while minimizing adverse outcomes. Future research must focus on establishing standardized protocols for NP characterization, enhancing their biodegradability and *in vivo* clearance, and devising strategies to circumvent the immune system’s recognition to prevent off-target effects. As we advance our understanding of NP interactions within biological systems, the prospects for their application in ischemic stroke therapy become increasingly tangible. The path forward will necessitate a collaborative effort across multidisciplinary fields to harness the full potential of nanomedicine in revolutionizing the management of ischemic stroke.

## Abbreviations

ATP: adenosine triphosphate
AuNPs: gold nanoparticles
BBB: blood–brain barrier
CBF: cerebral blood flow
CbNPs: carbon-based nanoparticles
CeONPs: cerium oxide nanoparticles
CNTs: carbon nanotubes
eNOS: endothelial nitric oxide synthase
GBNs: graphene-based nanomaterials
GO: graphene oxide
iNOS: inducible nitric oxide synthase
IR: ischemia–reperfusion
MRC: mitochondrial respiratory chain
MWCNTs: multi-walled carbon nanotubes
nNOS: neuronal nitric oxide synthase
NOS: nitric oxide synthase
NOX: NADPH oxidase
NPs: nanoparticles
PtNPs: platinum nanoparticles
rGO: reduced graphene oxide
RNS: reactive nitrogen species
ROS: reactive oxygen species
SeNPs: selenium nanoparticles
SOD: superoxide dismutase
SWCNT-PEG: PEGylated single-wall carbon nanotubes
US-FDA: United States Food and Drug Administration
XO: xanthine oxidase
